# Interplay of occupational stress, sense of humor, and health status among nurses working at hospitals in Ahvaz

**DOI:** 10.25122/jml-2020-0032

**Published:** 2021

**Authors:** Mahbubeh Babazadeh, Shahram Molavynejad, Ziba Parhamnia, Tahereh Boroun

**Affiliations:** 1. Nursing Care Research Center in Chronic Diseases, School of Nursing and Midwifery, Ahvaz Jundishapur University of Medical Sciences, Ahvaz, Iran; 2. Department of Nursing, School of Nursing and Midwifery, Ahvaz Jundishapur University of Medical Sciences, Ahvaz, Iran

**Keywords:** occupational stress, sense of humor, health status, nurse

## Abstract

Sense of humor constitutes a part of everyday life and work and an indispensable part of healthcare. However, the relationship between sense of humor and nursing occupational health and stress is yet to be studied in Iran. This study aimed to analyze the interplay of demographic factors, occupational stress, sense of humor, and health status of nurses working at the hospitals affiliated with the Ahvaz Jundishapur University of Medical Sciences, Iran. In this descriptive-analytical study, the statistical population consisted of 203 nurses. Occupational stress was assessed using the Iranian version of the Effort-Reward-Imbalance (ERI) Questionnaire. The Sense of Humor Questionnaire (SHQ) and the Iranian version of the 12-item General Health Questionnaire (GHQ-12) were employed to assess the sense of humor and physio-psychological health, respectively. Data collected were analyzed using the Statistical Package for the Social Sciences (SPSS) software. 81% (n=164) of the participants had an “external effort” (E) and “reward” (R) ratio greater than 1, indicating very high occupational stress. 39% (n=79) nurses with high occupational stress displayed their commitment to the workplace. Approximately 42% (n=85) of nurses were suffering from health problems. The mean humor score of participants was 2.90±0.41. Major life events over the past 3 months (p<0.01), weekly working hours (p<0.01), high occupational stress (p<0.01), and sense of humor (p<0.01) were determined to be significant predictors of nurses' health problems. Decision-makers are recommended to reduce nursing working hours through work shift management in order to maintain nurses' health status and reduce their occupational stress. In addition, hospital directors should promote a sense of humor in hospital environments with the help of culture-building practices, comedy books, music CDs, and training courses.

## Introduction

Occupational stress is defined as an individual’s physiological and emotional responses to an imbalance between job demands and abilities as well as resources and requirements. It is associated with negative health outcomes [1]. Nearly every occupation can cause stress [2]; however, the nursing profession is more likely to cause occupational stress because of a heavy workload, pain, patient death, sensitive interpersonal relationships, and low income [2, 3]. Nurses are not only responsible for their patients, but they also face a variety of difficulties, including reactions of patients’ family members, erratic work schedules, rules, procedures, and bureaucracy [2]. Accordingly, stressful work in the nursing profession is associated with increased levels of sickness absenteeism, poor physical and mental health, high turnover rates, and job abandonment. Workplace stress has been extensively studied in stressful occupations such as police work, education, and nursing. A study that included hospital nurses from Iran found that more than one-third of the participants experienced high levels of job stress and mental disorders [4]. Moreover, a review of the literature demonstrated the positive relationships of occupational stress, mental perceptions of psychological distress, and high incidence of stress-related occupational burnout and emotional exhaustion [5].

The coping strategy is a process of mitigating the effects of occupational stress on physio-psychological health in order to prevent distress, burnout, and mental disorders. Coping strategies can help to confront and adapt, enabling people to react to thoughts, behaviors, and emotions caused by occupational stress [6]. Coping strategies are classified as problem-solving coping and emotion-focused coping. Problem-solving coping is associated with techniques to minimize, redefine and/or solve external demands to reduce the effect of the stressor. Emotion-focused coping focuses on modifying negative psychological reactions to stress such as anxiety, fear, sadness, and anger [7]. The mitigating effect of the coping strategies is probably a function of the type of implemented mechanism. Coping responses are influenced by the source of stress, individual assessment, and workplace situations; for instance, nurses who suffer from occupational stress because of heavy workload use problem-solving strategies more frequently, whereas those who report patient demands and family-related conflicts as stressors use social support strategies [8, 9]. Research results have shown that efficient utilization of coping strategies reduced the level of stress and depression experienced [10]. Moreover, Chang *et al.* found that the avoidance coping strategy was significantly associated with physical and mental health [11]. This result means that occupational stress levels can be mediated if an individual performs effective coping strategies to handle the stressors. Interestingly, Ghiyasvandian and Gebra reported that coping strategies among hospital nurses in Iran are a well-accepted issue and have important positive outcomes in several areas of health [12]. Moreover, a study of hospital nurses in Iran found that among coping strategies, emotion-focused coping has been used more than problem-solving coping [13].

In recent years, a number of researchers have introduced humor as a coping mechanism for stress. Humor contributes to the alteration of an individual’s cognitive levels and outlook and may bring positive emotional change in their lives [14]. Humor has been negatively correlated with psychological problems, including depression, post-traumatic stress disorder (PTSD), anxiety, and stress in various studies [15–17]. In addition, several studies have demonstrated a positive correlation between humor, life satisfaction, self-esteem, optimism, happiness, and mental health [16, 18, 19]. These results suggest that although stress is associated with psychological distress, humor serves as a buffer for the individual against the harmful effects of stress [20].

From a physiological perspective, results have shown that laughter contributes to physical health by increasing heartbeat, enhancing circulation to vital organs, distributing nutrients, and improving chemical balance in the brain [14, 21]. Research has also shown that a good sense of humor leads to muscle relaxation, pain and discomfort control, positive moods, and overall mental health [20]. Laughter stimulates the secretion of endorphins and dopamine. These hormones reduce stress, relieve the sense of despair, reduce depression and reinforce positive thoughts. Therefore, laughing promotes health in humorous people by making them forget about the fear and anxiety of their stressful working environments [14, 22].

Since nurses spend a great deal of time with patients, humor can serve as an essential factor in the quality of their work and can affect their relationships within the medical team. It can also influence nurses’ satisfaction with their job when interacting with patients [22]. Therefore, nurses’ occupational stress might be reduced, and their health conditions might improve if they can create humorous situations in their workplace, something that subsequently enhances the quality of healthcare. A glance at the literature reveals that humor is used in a constructive style in certain countries, including the United States. In numerous healthcare settings, humor can serve as a factor to reduce workplace stress and maintain healthcare providers’ health [23]. However, no study that incorporated humor to explore the associations of occupational stress and humor with the health condition of the hospital nurses has yet been reported on this topic in Iran. Accordingly, this study aimed to analyze the relationship between socio-demographic factors, occupational stress, humor, and the health status of nurses working at the hospitals affiliated with the Ahvaz Jundishapur University of Medical Sciences, Ahvaz, Iran. The research findings will identify the predictors of the health status among hospital nurses and may provide hospital administrators with valuable ideas for maintaining nurses’ health status.

## Material and Methods

In this descriptive-analytical study, the research population consisted of 203 nurses working at the teaching hospitals affiliated with the Ahvaz Jundishapur University of Medical Sciences, Ahvaz, Iran. Sampling was performed from August to September 2020. Since regression analysis requires at least 10 participants per independent variable, the minimum number of participants had to be 160 because there were 16 independent variables in this study. According to past studies that included nursing staff, the return rate of the questionnaires was around 85–90% [14, 24]. Therefore, it requires a sample of 178–189 and 160 participants. Based on the inclusion criterion, there were 396 nurses in the teaching hospitals who could participate in the study. The inclusion criteria consisted of a minimum work experience of 6 months at teaching hospitals. During this time, we recruited 229 (57.8% of the whole population) nurses by stratified random sampling from different hospital units, including special units (emergency, operation, and hemodialysis room), critical care unit, medical unit, surgical unit, gynecology and obstetrics/pediatrics. Of a total of 229 nurses who were recruited, 203 nurses participated (response rate = 88.6%). Nurses who were excluded from the study were those who had failed to complete the questionnaires because of the leave of absence.

### Data collection instruments

Data collection instruments included a demographic questionnaire, the Iranian version of the Effort-Reward-Imbalance (ERI) Questionnaire, the Sense of Humor Questionnaire (SHQ), and the Iranian version of the 12-item General Health Questionnaire (GHQ-12).

• Demographic questionnaire: it included age, gender, educational attainment, educational continuation status, health status (whether the patient received a particular medication for a chronic illness), significant events during the past three months, ward, work experience, weekly working hours and the type of work shift;
• The Iranian version of the Effort-Reward-Imbalance (ERI) Questionnaire: The ERI Questionnaire was employed to measure occupational stress. The ERI Questionnaire contains 23 items, 6 of which measure effort, and 11 measure reward. The remaining 6 items measure over-commitment in individuals. The ERI Questionnaire was assessed on a 4-point Likert scale from “strongly agree” to “strongly disagree”. The imbalance between “effort” and “reward” was assessed by calculating the ratio of “external effort” (E) and “reward” (R) scores. An E/R≥1 ratio indicated that the work was done with great effort and low reward and that it was associated with high occupational stress. E/R≤1 ratios demonstrated low occupational stress. Individuals with very high scores were included in the “over commitment” parameter [14, 25];
• The Iranian Sense of Humor Questionnaire (SHQ): this tool was developed by researchers from Iran by reviewing Iranian articles [26, 27]. The questionnaire contains 30 items and four parameters of humor: a) attitude, b) creativity, c) perceptivity, and d) tendency. The SHQ was assessed on a 4-point Likert scale from “strongly disagree” to “strongly agree” with a score of 1–4. The individuals who obtained higher scores had greater senses of humor. Cronbach’s alpha coefficients for “humor attitude”, “ humor creativity”, “humor perceptivity” and “humor tendency” were 0.84, 0.89, 0.91, and 0.88, respectively;
• The Iranian version of the 12-Item General Health Questionnaire (GHQ-12): The 12-item General Health Questionnaire was developed by Goldberg in 1970. The internal consistency and validity of this questionnaire were assessed by Montazeri *et al.* in 2003 in Iran [28]. The questionnaire uses 12 items to assess individuals’ physical and mental health, focusing on measuring the pressures experienced by the body and mind as well as the social response. The higher an individual’s score, the lower their health. Each item is scored using a 4-point Likert scale. The “not at all” and “same as usual” responses were regarded as one category and received a score of zero. The “more than usual” and “even more than usual” responses were also classified under the same category and assigned a score of one. Those who received an overall score of 3 or more on this questionnaire were considered “cases”, i.e., having health problems, and those who received an overall score of 2 or less were considered “non-cases” [28].

### Analysis of results

Data were analyzed using the Statistical Package for the Social Sciences (SPSS), version 21. Demographic data were analyzed using descriptive statistics, including frequency, percentage, mean and standard deviation. Moreover, the Chi-square test was conducted to examine the relationship between participants’ health statuses (dependent variable) and categorical variables (independent variables). Student’s *t*-test and one-way ANOVA were employed to examine the differences between participants’ health statuses and continuous independent variables. Significant factors of participants’ health status were identified through multiple logistic regression analysis.

## Results

The sample included 203 nurses working at the hospitals affiliated with the Ahvaz Jundishapur University of Medical Sciences, Ahvaz, Iran. The majority of the participants were female (62.6%), and nearly 37.4% were male. The mean work experience was 7.68 years, and 93.6% of the nurses in this study did not receive medication for any chronic illnesses. In addition, nearly about 7.4% of them had experienced significant life events in the past 3 months (Table 1).

**Table 1.** Demographics of participants (n=203).

Variables	^Mean±SD^	^Number^	^Percentages^
**Age**	**33.14±8.45**	**203**	
**Sex**			
Male		76	37.4
Female		127	62.6
**Educational level**			
Bachelor		170	84
Master		33	16
Whether participating in continuing education		**203**	
Yes		34	16.7
No		169	83.3
Taking medicines for chronic diseases		203	
No		190	93.6
Yes		13	6.4
**Significant life events**		**203**	
No		188	92.6
Yes		15	7.4
**Working units**		203	
Medical units		51	25.1
Surgical units		22	10.8
Intensive care units		48	23.7
Gynecology and obstetrics/pediatrics		13	6.4
ER		19	9.4
OR		14	6.9
HD Room		36	17.7
**Years of experience**	**7.68±6.1**	203	
**Weekly working hours**			
<44 hr		118	58.1
45–47		66	32.5
≥48 hr		19	9.4
Shift type			
Day shift		29	14.3
Evening shift		11	5.4
Night shift		4	2
Three shifts		130	64
Day and evening shifts		29	14.3
**Job title**		**203**	
Head nurse		6	3
Associated head nurse		4	2
Team leader		6	3
Nurse		187	92

ER – emergency room; HD – hemodialysis room; OR – operating room.

### Occupational stress, humor, and nurses’ health

Results of the ERI questionnaire showed that the mean scores obtained by nurses for “external effort”, “reward” and “overcommitment” were 18.23±3.98, 40.84±8.01 and 17.1±3.01, respectively (Table 2). An E/R>1 ratio was obtained in 81% of cases, indicating very high occupational stress. These results show that the nursing job is done with great effort and poor reward. Seventy-nine nurses who endured high occupational stress stated that they had “overcommitted” to their job. Assessment of physio-psychological health showed that about 42% (n=85) of nurses had “health problems” (Table 2). Results from the assessment of the participants’ sense of humor showed a mean score of 2.90±0.41. In addition, the mean scores for the “humor attitude”, “humor creativity”, “humor perceptivity” and “humor tendency” parameters were 2.98±0.41, 2.86±.044, 3.18±0.39 and 2.78±0.49, respectively (Table 2).

**Table 2.** Scores and frequency distributions of occupational stress, social support, humor, and health status (n=203).

Variables	^Mean^	^(±SD)^	^Frequency^	^Percentage^
Extrinsic effort	18.23	3.98		
Reward	40.84	8.01		
Overcommitment	17.1	3.01		
Humor	2.90	0.41		
Attitude	2.98	0.39		
Creativity	2.86	0.44		
Perception	3.18	0.39		
**Tendency**	2.78	0.49		
**E/R ratio**				
>1			164	81
≤1			39	19
**Distribution of overcommitment**				
Top one-third			79	39
Last two-thirds			124	61
Health problems				
Yes			85	42
No			118	58

E/R ratio – external effort (E) and reward (R) ratio.

### The relationships of demographic characteristics, occupational stress, social support, humor, and nurses’ health

The research findings showed significant correlations in the participants’ health status, weekly working hours, and major life events in the past 3 months. There were no relationships between nurses’ health and their age, work experience, gender, educational attainment, educational continuation, wards, job title, medication for chronic illnesses, and type of shift (Table 3). Nurses’ health conditions were related to occupational stress (p<0.05) and overcommitment (p<0.05) to their jobs. Moreover, a significant positive relationship was observed between participants' health conditions and overall sense of humor (t=3.418, p=0.001), humor attitude (p=3.119, p=0.003), humor creativity (p=2.513, p=0.012), humor perceptivity (p=2.719, p=0.007) and humor tendency (p=2.931, p=0.047).

**Table 3.** Relationship between participants' health status and demographic characteristics, occupational stress, social support and humor.

Variables	No health problems (n=118)	Mean (±SD) n (%)	With health problems (n=85)	Mean (±SD) n (%)	X^2^	P-value
**Age**	33.11	8.75	33.17	8.12	0.038	0.962
**Sex**						
Male	70	92	6	8		
Female	110	86.6	17	13.4		
**Educational level**						
Bachelor	148	87.05	22	12.95	0.619	0.816 (f)
Master	30	90.9	3	9.1		
**Whether participating in continuing education**						
Yes	30	88.2	4	11.76		0.315 (f)
No	137	81.6	32	18.9		
**Taking medicines for chronic diseases**						
No	152	79.16	40	20.8		0.088 (f)
Yes	24	94.1	10	5.9		
**Significant life events**						
No	111	59.04	77	40.96		0.031 (f)
Yes	11	73.3	4	26.7		
**Working units**						
Medical units	55	90.16	6	9.84	1.613	0.963 (c)
Surgical units	18	81.81	4	18.19		
Intensive care units	37	77.08	11	22.92		
Gynecology and obstetrics/pediatrics	10	76.92	3	23.07		
ER	15	78.95	4	21.05		
OR	13	92.85	1	7.15		
HD Room	27	75	9	25		
**Years of experience**	149	69.6	65	30.4	1.812	0.094 (t)
**Weekly working hours**						
<44 hr	98	83.05	20	16.95		0.312 (f)
45–47	54	81.81	12	18.19		
≥48 hr	14	73.7	5	26.3		
**Shift type**						
Day shift	25	86.2	4	13.8	1.812	0.734 (c)
Evening shift	11	100	0	0		
Night shift	4	100	0	0		
Three shifts	104	80	26	20		
Day and evening shifts	25		4			
**Job title**						
Head nurse	5	83.3	1	16.7	1.695	0.649 (c)
Associated head nurse	4	100	0	0		
Team leader	5	83.3	1	16.7		
Nurse	154	82.35	33	17.65		
**E/R ratio**						
>1	140	85.36	24	14.64		0.005 (f)
≤1	31	79.48	8	20.52		
**Distribution of overcommitment**	126	62.06	77	37.94		0.016 (f)
Top one-third	60	75.94	19	24.06		
Last two-thirds	106	85.48	18	14.52		
**Overcommitment**	15.5	2.43	16.9	2.214	-4.689	1.198 (t)
**Humor**	2.893	0.401	2.92	0.391	3.418	0.001 (t)
**Attitude**	2.862	0.409	2.721	0.461	2.513	0.012 (t)
**Creativity**	2.851	0.409	2.721	0.461	2.513	0.012 (t)
**Perception**	3.218	0.298	3.043	0.473	2.719	0.047 (t)
**Tendency**	2.834	0.512	2.712	0.509	2.931	0.008 (t)

E/R ratio – external effort (E) and reward (R) ratio; ER – emergency room; HD – hemodialysis room; OR – operating room.

### Predictors of nurses’ health status

The results of the multivariate analysis identified such variables as weekly working hours, major life events over the past 3 months, high occupational stress, and humor as significant predictors of nurses’ health problems (Table 4). The results showed that nurses who worked over 48 hours per week suffered from health problems 4.28 times (Exp (B)=4.28; p<0.01) more than those who worked less than 44 hours per week. In addition, nurses who experienced major life events during the past 3 months were 3.89 times more likely to develop health problems (Exp (B)=4.28; p<0.01) than other nurses (Table 3). Nurses with an E/R≥1 ratio (high occupational stress) suffered from health problems 3.124 times more than those with an E/R≤1 ratio (Table 3).

**Table 4.** Significant predictors of participants' health.

Variables	B	SE	Wald	P-value	Exp (B)
**Weekly working hours (≥48 hr)**	1.272	0.412	8.12	0.003	4.282
**Any significant life events (Yes/No)**	1.298	0.498	7.32	0.001	3.891
**Work stress**					
**E/R ratio (>1/≤1)**	1.112	0.326	11.31	0.002	3.124
**Overcommitment (top one-third/last two-thirds)**	1.003	0.29	10.9	0.001	2.817
**Mean scores of humor**	-1.39	0.49	9.34	0.001	0.229
**Constant**	1.735	1.46	1.301	0.259	5.415

E/R ratio – external effort (E) and reward (R) ratio.

## Discussion

In this study, the significant predictors of nurses’ health included weekly working hours, major life events during the past 3 months, high occupational stress, and humor. The results of this study are in line with the findings of Fang *et al.* (2019) [14]. The problem of nurses’ health, which has a major contribution to their performance, has turned into one of the main concerns of all healthcare administrators. Therefore, understanding the factors related to nurses’ health may prove helpful in promoting their health and, subsequently, improving the quality of patient care [13]. The research results showed a positive correlation between the health status of nurses with “weekly working hours” and “major life events during the past 3 months”. This result is consistent with the findings of Smith *et al.* [29]. Nurses have to perform significant tasks such as transporting patients and frequent manual tasks during working hours. In addition to extensive efforts, the nursing profession demands enormous accountability. Therefore, over time, nursing duties result in inadequate relaxation of the body and mind, which may eventually lead to health problems such as musculoskeletal disorders [30]. The research findings are consistent with those of Smith *et al.* [31]. In addition, major life events such as the death of loved ones, divorce, or certain everyday troubles may endanger the health of various groups in society, including nurses, and may affect their work responsibilities [5]. Similarly, and in line with the findings of Fang *et al.*, major life events over the past 3 months were significantly correlated with the health status of the nurses participating in this study [14].

The research findings showed an E/R>1 ratio in 81% of nurses, signifying that nurses’ great efforts were accompanied by poor rewards (high occupational stress), which can easily lead to health problems in nurses. The research results support the findings of Fang and Hung, who showed that nurses working under high occupational stress at hospitals were more likely to have health problems [30]. Humor has been found to be very useful in improving and maintaining health in the face of stressful working conditions such as nursing [32]. In their study, Fang *et al.* demonstrated significant positive relationships among humor attitude, creativity, and perceptivity [14]. Consistent with studies of Feldman and Steptoe, Loukzadeh and Bafrooi and Fang *et al.*, the results of the present study showed a significant positive relationship between all humor parameters and the health status of nurses [13, 14, 33]. In fact, there is evidence suggesting the stress-relief effects of humor based on mental health outcomes in healthy individuals and patient populations [32, 34]. Humor is associated with poor cardiovascular response to hospital stressors and enhanced immune function, both of which may affect physical health [35]. In addition, there are numerous psychological benefits to humor. Analysis of nurses’ memories revealed that using humor would help nurses and patients cope with the unpleasant situations they might find themselves in at work [36]. In cross-sectional and self-report studies conducted on different populations, humor was shown to be associated with low anxiety, depression, and dysphoria levels [36]. Hormones such as endorphins, serotonin, and dopamine are released from endocrine glands in response to humor-induced laughter. These hormones promote positive thinking and creativity and reduce the signs of depression. Therefore, humorous nurses can potentially maintain their health status and easily perform their daily tasks at the hospital [37]. In fact, humor allows the nurse to adapt to long, arduous, and emotional working hours and encourages open communication and team spirit. Therefore, humor can be used as an effective coping strategy in stressful settings such as hospitals [38]. Consistent with the results of this study showing a positive relationship between humor and nursing health status, numerous studies of healthcare providers show that not only do nurses who use humorous communications under conditions of general stress actively use humor as a coping mechanism, but they also show up to the workplace with greater enthusiasm and deliver a more effective performance [39]. Regarding the limitations of the study, participants in the study had only been selected from a few hospitals in one city; therefore, the sample may not be an adequate representative of the nursing population. Further studies are required to examine the generalizability of research results to nurses working at other hospitals. Another limitation lay in the use of self-reported measures and the cross-sectional design. Future studies should improve the reliability and validity of this study through biological assays, including blood pressure measurement, detection of coronary artery diseases, and salivary cortisol levels as health indices. Some variables such as bed capacity, daily admission, and mortality rate, which may affect the results, were not included in the present study.

## Conclusion

The research results showed that nearly 81% of nurses working at the hospitals affiliated with the Ahvaz Jundishapur University of Medical Sciences suffered from high occupational stress, meaning that nursing tasks were done with great effort and poor reward. Nearly 42% of nurses also suffered from health problems. Nurses’ health problems in this study were related to weekly working hours, major life events over the past 3 months, and humor. Major life events over the past 3 months, weekly working hours, high occupational stress, and humor were determined to be significant predictors of nurses’ health problems. According to the research findings, it is recommended that hospital administrators develop policies for work shift planning to prevent nurses from working over 48 hours per week in order to maintain the health status of nurses. In addition, it is necessary to provide nurses with training courses on stress management, humor culture-building practices for stress relief, and implement coping strategies against stress.

## Acknowledgments

### Ethical approval

The approval for this study was obtained from the Ethics Committee of the Ahvaz Jundishapour University of Medical Sciences, Ahvaz, Iran (approval ID: IR.AJUMS.REC.1399.446).

### Consent to participate

Written informed consent was obtained from the participants.

### Conflict of interest

The authors declare that there is no conflict of interest.
